# Impact of the implementation of an online network support tool among clinicians of Primary Health Care and Specialists: ECOPIH Project

**DOI:** 10.1186/1471-2296-14-146

**Published:** 2013-10-03

**Authors:** David Lacasta Tintorer, Souhel Flayeh Beneyto, Xavier Alzaga Reig, Xavier Mundet Tuduri, Josep Anton De la Fuente, Josep Maria Manresa, Pere Torán Monserrat, Francesc Saigí Rubió

**Affiliations:** 1 Centre d’Atenció Primària la Salut. Institut Català de la Salut, Passatge dels Encants s/n, 08914, Badalona, Spain; 2 Unitat de Suport a la Recerca Metropolitana Nord. IDIAP Jordi Gol, Carrer Major 49-53, 08921, Santa Coloma de Gramenet, Spain; 3 Universitat Autònoma de Barcelona, Plaça Cívica, 08193, Bellaterra, Cerdanyola del Vallès, Spain; 4 Centre d’Atenció Primària Dr Robert. Institut Català de la Salut, Plaça de la Medicina s/n, 08911, Badalona, Spain; 5 Unitat de Suport a la Recerca Barcelona Ciutat. IDIAP Jordi Gol, Carrer Sardenya 375, 08025, Barcelona, Spain; 6 Departament de Ciències Mèdiques, Universitat de Girona, Carrer Emili Grahit 77, 17071, Girona, Spain; 7 Universitat Oberta de Catalunya, Carrer Roc Boronat 117, 08018, Barcelona, Spain

**Keywords:** Remote consultation, Primary health care, Problem solving, Telemedicine, Referral and consultation, Education medical continuing

## Abstract

**Background:**

There has been created an online communication tool with the objective to improve the communication among different levels of care, between Primary Care clinicians and Specialists. This tool is web 2.0 based technology (ECOPIH project). It allows to review clinical cases and to share knowledge. Our study will evaluate its impact in terms of reduction on the number of referrals to three specialties two years after the use of this tool.

**Methods/Design:**

Open, multicenter, controlled, non random intervention study over 24 months. Study population includes 131 Primary Care Physicians assigned to nine health centers. The study will compare the clinicians that use the ECOPIH with the ones that do not use the tool. Also, professionals that start to use the tool during the period time of the study will be included.

The number of annual referrals during the first and second year will be analyzed and retrospectively compared with the previous year to the implementation of the tool. Moreover, it will be assessed the level of satisfaction of the professionals with the tool and to what extend the tool responds to their needs.

**Discussion:**

The implementation of ECOPIH in the field of Primary Health Care can decrease the number of referrals from primary care to specialist care.

It is expected that the reduction will be more noticeable in the group of professionals that use more intensively the tool. Furthermore, we believe that it can be also observed with the professionals that read the contributions of the others.

We anticipate high degree of customer satisfaction as it is a very helpful resource never used before in our environment.

## Background

The medical activity is associated with intensive management of information and generation of knowledge. The communication among professionals and team work are essential to the professional development. Nowadays, this is sustained by information and communication technologies (ICT). In the globalization framework the adoption and efficient use of ICT by health professionals is linked to the professional competition. ICT has created great expectation as a tool to face the challenge of socioeconomic changes in the health system of the 21^st^ century [1].

In numerous health areas the creation of free information networks and strategies has been paramount to the development of new knowledge to confront the continued innovation of the medical activity [2]. Recently, new technologies, applications and services have appeared in Internet. They are Web 2,0 elements that go for creativity, apomediation, aggregation and share of information (not only for scientists) and collaboration through social networks, wikis or blogs among others. Internet users, geographically scattered, are organized around communities of common interests giving room to non-formal or unregulated learning.

In everyday clinical activity primary health care doctors face numerous uncertainties, between 0,7 and 18,5 questions per 10 patients [3]. Clinical sessions and individual conversations (in person and over the phone), together with specialist care, are options that allow such issues to be resolved. On the other hand, the learning capacity of doctors improves when they confront real problems in their daily activity that do not represent long time investment [3,4]. Given that the health system is at saturation point, communication between primary and specialist care is not easy, quick or effective, and it leads to many referrals to specialist care (hospitalisation or specialist consultations) that generally entail excessive delays for appointments [5].

Given these communication difficulties [6], several telemedicine approaches have been tried out in recent years. Of these, e-mail and videoconferencing [5,7,8] have proven to be of particular benefit in terms of efficiency, cost-effectiveness and improved medical care [9]. A number of studies that have assessed healthcare professionals’ levels of satisfaction with the use of telemedicine platforms applied to information and communication have produced good results in terms of improved medical care and use of time [10-12].

Newer still is the creation of communities of practice in the field of healthcare [13]. These are online platforms that draw on the advantages of Web 2.0 to build knowledge among healthcare professionals working at different levels of care [14]. These virtual communities have proven capable of solving healthcare professionals’ information and communication problems in a much simpler way. Moreover, they have been found to improve the functioning of organisations [13] by generating the kind of tacit knowledge that emerges from interactions among colleagues [14,15].

We could not find any study that assesses this issue at the level of a primary care area and its different health centers in the long run. On the other hand, the effectiveness of Community of Practice (CoP) on the functioning of health organizations has not been evaluated in a proper way [16].

**Figure 1:**
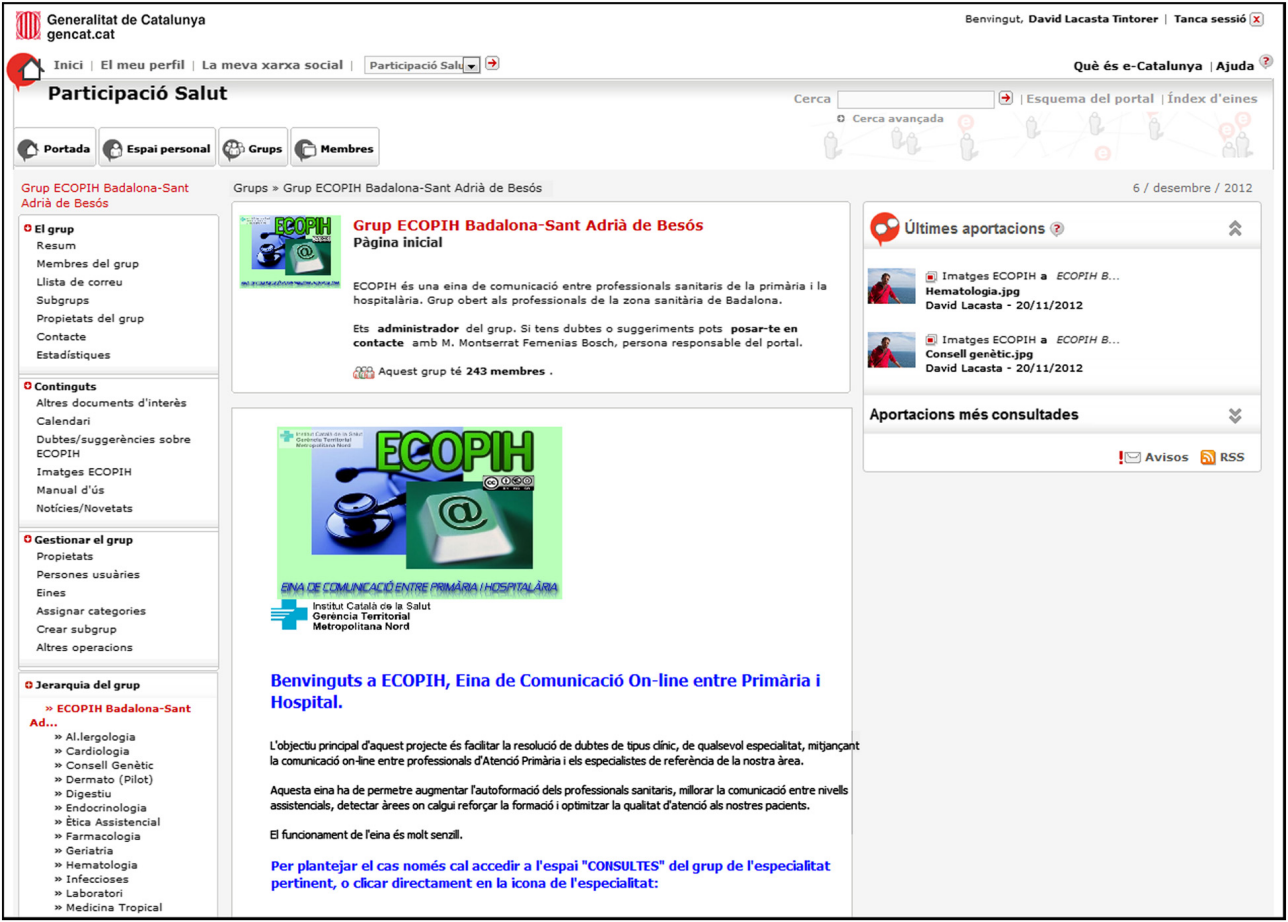
Home page of ECOPIH.

Due to the expansion and emerging interest that ICT has generated in the society, ECOPIH project (Online Communication Tool Between Primary and Hospital Care) was created. It lies in the design and implementation of a virtual network with web 2.0 technology that allows interaction and communication among health professionals (doctors and nurses) of primary care and specialist care (Figure 1). Our aim is to design a study that assesses the efficacy of this tool to decrease referrals from primary care to specialist care and hospitals.

## Methods/Design

### Hypothesis

#### ***1^st^ Hypothesis***

Primary care professionals confront more everyday with patients with multiple pathologies and chronic processes. This has dramatically increased the complexity of health care. They are supported by specialist care through hospitalization or referral to the specialist consultation. The implementation of ICT tool ECOPIH with fluid flow of information between the two levels of care and promoting the creation of virtual communities would decrease significantly the number of referrals from primary care to the clinical specialties of Cardiology, Endocrinology and Gastroenterology.

#### ***2^nd^ Hypothesis***

The use of the ECOPIH tool would increase primary care clinician satisfaction and improve their ability to manage chronic patients.

### Objectives

#### ***General objective***

To assess the impact of the ECOPIH on the number of referrals from a primary health care area to the specialist care of Cardiology, Endocrinology and Gastroenterology.

#### ***Specific objectives***

1. To compare the number of referrals from the primary care clinicians that use ECOPIH with the clinicians that do not use it.

2. To determine if there is a connection between the frequency on the use of the ECOPIH and the reduction of the referral rate for each clinician.

3. To evaluate the referral rate between the clinicians that inquiry about clinical cases with the clinicians that only consult their colleague’s contributions.

4. To analyze the frequency of utilization of ECOPIH according to the different features of the participants: age, gender, health center, number of inquiries, number of contributions and number of clinical consultations.

5. To evaluate the level of satisfaction among clinicians using ECOPIH.

### Design

Open, multicenter, controlled, non random intervention study over 24 months of follow up.

The study will be carried out in a Health Service Area of Barcelona Province with 9 primary health care centers and 624 health staff that server 227.151 population. All clinicians of the Primary Care System will be analyzed (131 physicians). All facilities and staff belong to the public health system of Catalonia (Spain).

#### ***Inclusion criteria***

Clinicians of the primary care area (SAP Badalona Sant Adrià) of the study that are working at least one year in the same health center.

#### ***Exclusion criteria***

Pediatricians of primary care.

Other non-clinician staff.

#### ***Sample size***

64 clinicians out of the 131 working in the area joined up the ECOPIH network since 1^st^ July 2011 (ratio ECOPIH / non ECOPIH 0.955). Average referral rate per clinician is 33.5 (SD 8.0) per 100 patients that consulted in 2010. With a bilateral level of significance of 5% and power 80% a sample of 120 clinicians would allow to detect an interaction at 2 levels (ECOPIH / non-ECOPIH) with a minimum difference of 1.5977 units in a variance analysis for repeated measures, assuming a correlation coefficient between two times measures of 0.1 and a participants ratio (ECOPIH / non-ECOPIH) of 1:1, 2:1 or 3:1. (StudySize 2.0 was used for calculations) [17].

#### ***Evaluation of the ECOPIH platform***

The ECOPIH project is a web 2.0-based virtual network that allows online interaction and communication among health professionals (physicians and nurses) from primary and specialist care. The tool is used to seek advice for clinical cases with the specialist. The inquires are available to all members of the network and they can contribute to the discussion. Also, it is possible to attach any document related to the issue that is considered of interest.

The platform e-Catalunya (http://ecatalunya.gencat.cat) was selected as Content Management System because offers directly a great number of applications, incremental deployment options and integration with well-known environments, which enables users to quickly share information and easy. It also lets you work with corporate level standards that provide security, privacy, management and integration into a stable platform and closed. It includes a forum (“Consultation”), a space to attach documents (“Documents”) and images (“Images”) and a blog (“News / Novelties”) where everyone can add comments and get information about other groups. It also has a tool (“Participation”) that allows to make surveys among participating members, as well as a calendar (“Calendar”) and a tool for online document editing (“e-Wiki”). The platform has other features that were deactivated to simplify it use.

**Figure 2:**
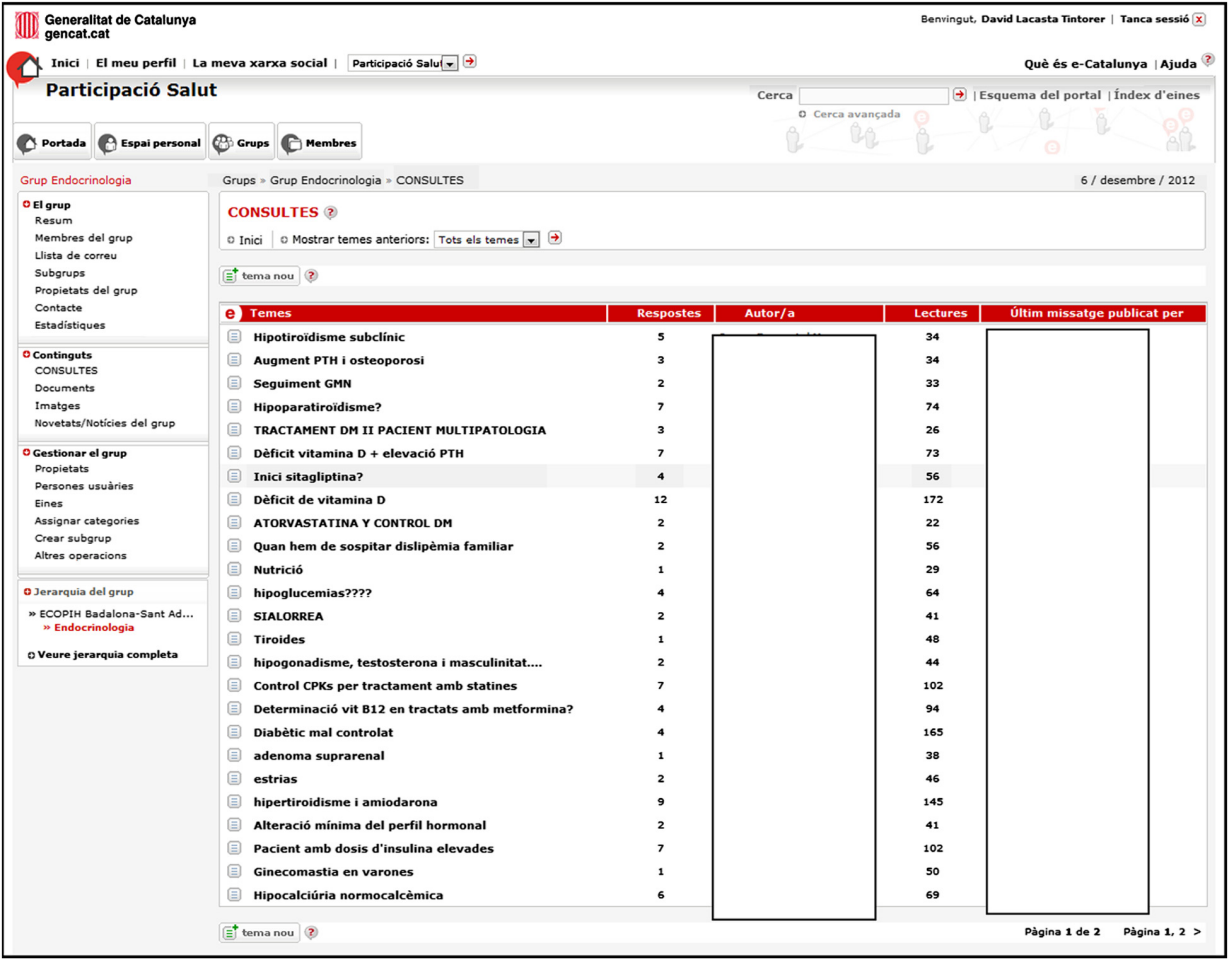
Consultations Forum.

**Figure 3:**
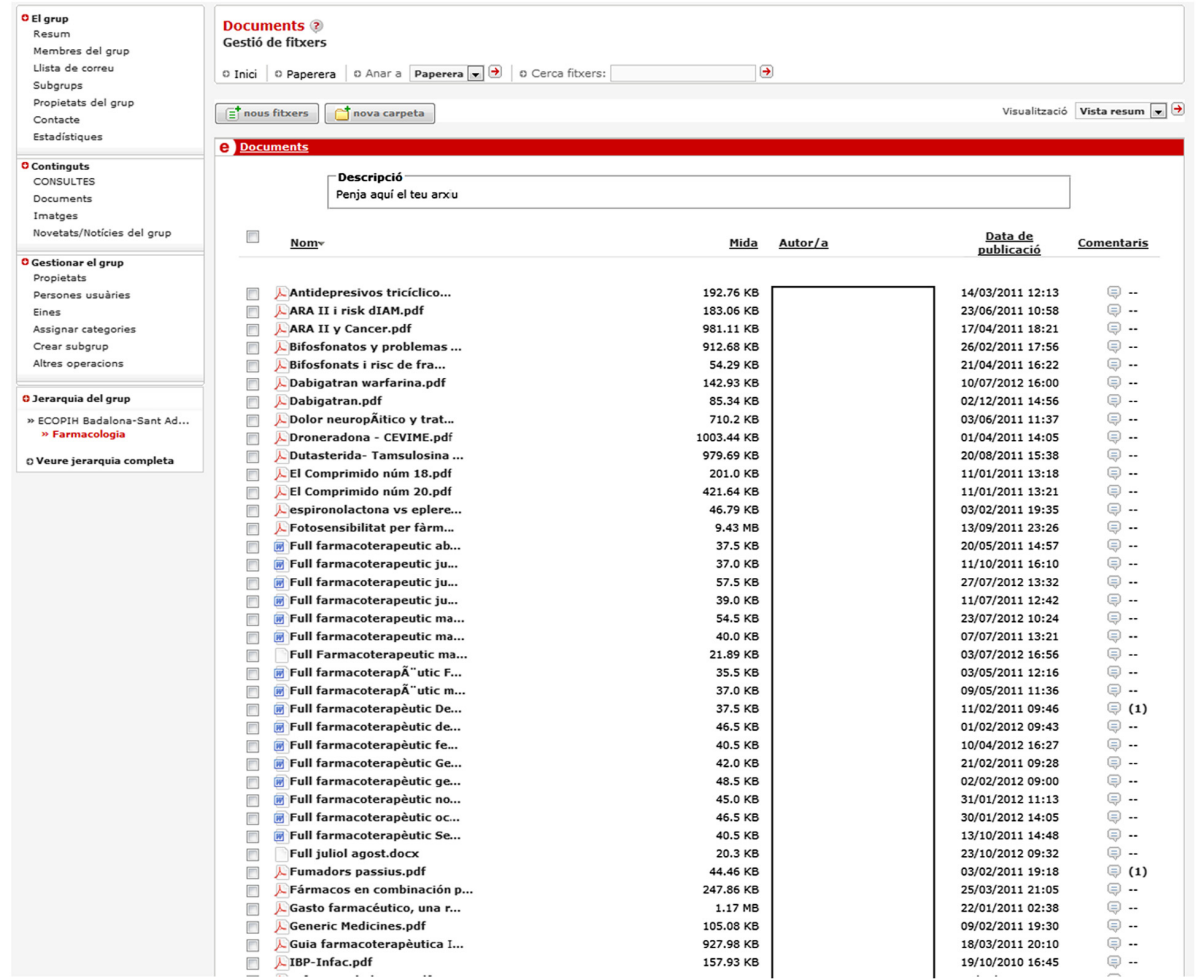
Document repository.

The mentioned platform is adapted to the particular needs of the network tool. When a clinician wants to inquire about a clinical case it has to write it in the forum providing protocols or bibliography about the case if necessary (Figure 2). Can also provide documents (Figure 3) that illustrate the case (photos, x-ray images, clinical reports, electrocardiograms). The referral specialist answers the issues raised and provides literature or clinical protocols that expand their response if necessary. The rest of the primary care members can read the documents and participate in the discussion as well as sharing documents or contributing to the blog. Participation of primary care clinicians and nurses is voluntary. Documents can be searched using various attributes and document content.

The head of each specialty department was contacted in order to present the project and to explain the role of the participant in the ECOPIH as a consultant (to reply queries and attach interesting documents) being important that the process does not take more than 48 hours.

With these contacts with the heads the specialties involved have increased substantially being nowadays 30 active specialties. Among them Cardiology, Palliative Care, Dermatology, Endocrinology, Pharmacology, Gastroenterology, Laboratory, Pneumology. It is expected to include all the surgical and medical specialties of the hospitals. That will represent an important contribution to the integration of different public institutions of the health sector.

Patient confidentiality is a key issue and it is taken into account to run ECOPIH. Only the age and gender will be recorded.

The use of this tool is strictly of an informative nature, the family doctor is in charge and responsible for the patient. The specialist will not be accountable for the adopted decisions.

#### ***Study arms***

The clinicians will be grouped in three arms:

1. First will include all clinicians assigned to ECOPIH network before 1^st^ July 2011.

2. The second will be the control group of the first and will include all clinicians not attached to ECOPIH before 1^st^ July 2011.

3. The third group will include all professionals not enrolled to ECOPIH before 1^st^ July 2011 but registered in the network at any time during the study period. They should be in the network for at least 12 months.

#### ***Study variables***

##### 

**Main variable** Number of cumulative referrals per assigned / not assigned clinician to ECOPIH platform within 12 months prior to their application and 12 and 24 months after the implementation of ECOPIH.

##### Secondary variables

– Concerning the clinicians: Gender, age (date of birth), health facility, application to ECOPIH (yes/no), referral specialist, number of consultations/connections to the network, active participation (write inquiries or answer messages).

– Concerning the network: overall number of members, number of members by specialty, number of inquiries to the community, number of contributions, number of issues raised, day of the week and time of the inquiry and/or contribution.

– A specific anonymous questionnaire will be distributed to the participants to assess user satisfaction and whether the tool is user friendly.

#### ***Statistical analysis***

It will be analyzed if there is a association between participating in the ECOPIH network and the evolution of the mean percentage of referrals by 100 visited patients. It will be considered a significant difference of 1.57 units. ANOVA Repeated Measures (One within factor)will be used to compare basal values (12 months before) with data obtained between 1 to 24 months after.

It is foreseen to do a descriptive analysis of the two group variables. Qualitative variables will be described in absolute and relative frequencies. Quantitative variables will include mean and standard deviation. If there is skewed distribution then median and quartiles will be used. Confidence interval will be 95%.

To compare proportions Pearson, Fisher or Chi square tests for lineal trend will be used. To compare quantitative variables t- Student test will be used for independent or paired data. In case of skewed distribution U Mann Whitney, Wilcoxon or McNemar tests will be used according to the conditions of application.

Level of significance will be p ≤ 0.05. Statistical analyses will be performed using the SPSS v.18for Windows 18.0.

### Ethics

Confidentiality will be preserved at all levels ensuring that professionals and patients will not be identified. From ECOPIH platform is not possible to access the medical history of patients. Information will be obtained from existing data and subject identifying information will be coded and anonymized. Consequently, there is no need for informed consent.

This project follows the national regulation (Spanish Law 14/2007, 3^rd^ July, Biomedical Research) and International regulations for ethical issues (Helsinky and Tokio declarations). The features of the intervention exclude the study to meet the national regulations for clinical trials. On the other hand, confidentiality is guaranteed under the Personal Data Protection Law (15/1999, 13^th^ December). All participants will be in-writing informed about their participation on the study. No information about the intervention will be provided to avoid bias.

The research protocol has been reviewed and approved by the Ethics Committee and Clinical Research of the Primary Care Research Institute IDIAP Jordi Gol (Barcelona, Spain).

## Discussion

The inclusion of ICT in the field of health care, specifically applied to the communication among professionals at different levels of care, should improve better coordination among professionals and decrease the number of referrals from primary to specialist care. The integration of web 2.0 concept allows the community members to provide contents and to share knowledge improving the communication among professionals. Therefore their clinical practice improves by learning form others’ experiences and updating their knowledge.

For this reason a tool has been designed to allow the discussion of clinical cases from more than 30 specialties. It is also a reservoir of documents and has a blog where information can be shared and updated.

ECOPIH enables the transmission of knowledge among health professionals to solve doubts regarding actual clinical situations and is also becoming a collaboration and continuous training to maintain updated knowledge. The project has raised much interest in other primary health care regions of Catalonia. A nearby region has already implemented it, and other two will do it the near future. The management of ECOPIH will be uniform in all regions but the everyday running will be independent as each region has its own reference specialists, except, for five specialties: Smoking, Ethics, Immunization/Vaccines, Ulcers and Podiatry that have the same references for overall Catalonia.

It’s important to analyze the impact of the implementation of this kind of web 2.0 tools on the reduction of the number of referrals, as well as to assess the level of interest from the professionals to this technology and their preferences and opinions in order that they can be part of the tools commonly used in primary care.

There are some limitations to the study. There is the possibility that many professionals register to ECOPIH during the follow up period. We have foreseen the development of an “informative campaign” to attract all hesitant professionals that would decide to register later on. The analysis at 24 months will include clinicians with at least 12 months in the network. Furthermore, in the sample size calculation we foresee the participation ratio of ECOPIH / non- ECOPIH from 1:1 to 3:1.

It is highly unlikely that all clinicians register to ECOPIH network (it is voluntary). In this case the analysis would evaluate the whole clinician group and the clinicians according to the level of utilization of the network.

Could be the bias that professionals working with ECOPIH platform are people especially motivated by some aspect of clinical practice that makes their referrals to specialists be made in a differentiated manner. This bias would affect the analysis in this group. We have foreseen to analyze the reduction of referrals of each professional related to the ones before the implementation of the study. Hence, this possible bias would be controlled by the study design.

The number of referrals from a specific area can be affected by several factors like difficulty to request additional tests, long waiting lists or lack of time to use the tool. However this will affect both arms of the study and will be controlled by the analysis.

We foresee the following consequences of the intervention:

– To take advantage of the communication among clinicians incrementing the organization’s knowledge.

– Better communication among professionals and optimization of the utilization of health resources.

– To include web 2.0 tools in daily practice of primary care professionals.

– To improve continuing medical training and clinical competence of practitioners to encourage self-learning based on problems linked to practice.

If it is confirmed that the implementation of a ECOPIH tool in a primary health care area will improve clinicians knowledge this will have repercussions on better care to patients. Patients would have virtual access to their cases. The improvement of the resolution of clinical cases at primary level will end on the reduction of the number of referrals. If the project confirms the expected results then we will have a collaborative working system among clinicians that will benefit at three axes: better quality of health care, better professional competency and empowerment, and finally improvement on the efficiency of the health system.

Therefore, we think that the obtained results will prove a valuable knowledge for a better planning and utilization of health resources and professional practice.

## Abbreviations

CoP: Community of Practice; ECOPIH: Eina de Comunicació entre Primària i Hospitalària (Online Communication Tool between Primary and Hospital Care); ICT: Information and communication technologies; PCP: Primary care providers; PHC: Primary Health Care; SAP: Servei d’Atenció Primària (Primary Health Service Area).

## Competing interests

The author(s) declare that they have no competing interests.

## Authors’ contributions

All the authors have had a substantial contribution to the research design and study protocol. DLT (ECOPIH Community Manager) is the coordinator of the investigation and has participated in the elaboration of the protocol of investigation and the writing of this article. Also involved in the collection and analysis of results. SFB (ECOPIH Community Manager) participation in the drafting of the study protocol, data collection and dissemination of results. FSR contributed to formulating the research question, study design, interpretation of results and drafting the article. Expert in telemedicine and health 2.0 tools XAR participation in the study design, literature search, interpretation and dissemination of results. Expert in Health 2.0 tools. XMT protocol design, interpretation of results, drafting the article. JAF methodology and protocol design, data manager, interpretation of results, drafting the article. JMM supervised the methodology of the protocol of investigation and will be responsible for the treatment of the data and statistical analysis. PTM contributed to formulating research question, study design, analysis and interpretation of results. All the authors have read, revised and approved the final manuscript.

## Pre-publication history

The pre-publication history for this paper can be accessed here:

http://www.biomedcentral.com/1471-2296/14/146/prepub
